# Multi-Objective Optimization of Nutritional, Environmental and Economic Aspects of Diets Applied to the Spanish Context

**DOI:** 10.3390/foods9111677

**Published:** 2020-11-16

**Authors:** Ricardo Abejón, Laura Batlle-Bayer, Jara Laso, Alba Bala, Ian Vazquez-Rowe, Gustavo Larrea-Gallegos, María Margallo, Jorge Cristobal, Rita Puig, Pere Fullana-i-Palmer, Rubén Aldaco

**Affiliations:** 1 Department of Chemical and Biomolecular Engineering, University of Cantabria, Avda. De los Castros, s.n., 39005 Santander, Spain; ricardo.abejon@unican.es (R.A.); jara.laso@unican.es (J.L.); margallom@unican.es (M.M.); jorge.cristobal@unican.es (J.C.); 2 Departamento de Ingeniería Química, Universidad de Santiago de Chile. Av. Libertador Bernardo O’Higgins 3363, Estación Central, Santiago 9170019, Chile; 3 UNESCO Chair in Life Cycle and Climate Change ESCI-UPF, Pg. Pujades 1, 08003 Barcelona, Spain; laura.batlle@esci.upf.edu (L.B.-B.); alba.bala@esci.upf.edu (A.B.); pere.fullana@esci.upf.edu (P.F.-i.-P.); 4 Peruvian LCA Network (PELCAN), Department of Engineering, Pontificia Universidad Católica del Perú, Av. Universitaria 1801, San Miguel, Lima 15088, Peru; ian.vazquez@pucp.pe; 5 Environmental Research and Innovation (ERIN), Luxembourg Institute of Science and Technology, Esch-sur-Alzette, 4362 Luxembourg, Luxembourg; gustavo.larrea@list.lu; 6 Department of Computer Science and Industrial Engineering, Universitat de Lleida (UdL), Pla de la Massa, 8, 08700 Igualada, Spain; rita.puig@udl.cat

**Keywords:** diets, sustainability, nutrition, environmental impact, economic costs, life cycle assessment

## Abstract

Current food consumption patterns must be revised in order to improve their sustainability. The nutritional, environmental, and economic consequences of these dietary patterns must be taken into consideration when diet guidelines are proposed. This study applied a systematic optimization methodology to define sustainable dietary patterns complying with nutritional, environmental, and economic issues. The methodology was based on a multi-objective optimization model that considered a distance-to-target approach. Although the three simultaneous objectives (maximal nutritional contribution, minimal greenhouse gas emissions, and minimal costs) could be divergent, the proposed model identified the optimal intake of each food product to achieve the maximal level of nutritional, environmental, and economic diets. This model was applied to six different eating patterns within the Spanish context: one based on current food consumption and five alternative diets. The results revealed that dietary patterns with improved nutritional profiles and reduced environmental impacts could be defined without additional costs just by increasing the consumption of vegetables, fruits, and legumes, while reducing the intake of meat and fish.

## 1. Introduction

Food production contributes significantly to climate change and other environmental impacts [1], and further contribution is expected with the projected increase in food demand by 2050. In this regard, human diets, the main driver of food production, play a crucial role. If eating patterns keep globally shifting towards a Western diet, with larger intakes of meat and processed food products, current food-related environmental impacts can increase by 80% in 2050 [2]. Hence, food consumption optimization is a key measure for climate change mitigation.

A considerable amount of scientific research has recently focused on analyzing the potential environmental benefits of diet shifts. Most research [3,4,5,6] define dietary scenarios based on nutritional guidelines (i.e., the national dietary guidelines, the Mediterranean or vegetarian diets), apply the Life Cycle Assessment (LCA) [7], to assess the environmental impacts, especially the greenhouse gas (GHG) emissions [8], and compare them to the ones of current eating habits. However, defining these scenarios is challenging since their accuracy will depend on the degree of detail of these guidelines regarding the recommended quantity of food ingredients, as shown by Springmann et al. [9]. In this regard, optimization can be a useful tool to support the definition of dietary guidelines [10,11] and sustainable diets, since it can consider multiple aspects—environmental, nutritional, and economic [12].

Linear programming (LP) is the most common method applied to optimize diets. LP is a mathematical modeling that optimizes an objective function—a linear equation—that must follow certain constraints (i.e., nutritional). Macdiarmid et al. [13] were the first to apply LP on the environmental sustainability of diets together with health. In this study, they reported a nutritious and affordable diet with 36% lower GHG emissions. Thereafter, some studies [14,15,16] minimized the environmental impacts, following Macdiarmid’s methodology. Donati et al. [17] also followed Macdiarmid’s approach, but they applied a multi-objective target programming (MTP) approach by optimizing simultaneously three environmental impacts together with the cost of diets (Table 1) Other LP studies in diets [12,18] minimized the total difference to the baseline diet by applying quadratic programming (QP); transforming the non-linear function to a linear one following the method used by Darmon et al. [19].

However, few studies on diet optimization apply non-linear programming (NLP) or more complex optimization formulations. As far as we were able to ascertain, the study of Chaudhary and Krishna [20] is the only one that has used a non-linear optimization algorithm to optimize the difference from a baseline diet (Table 1), with 38 constraints, 5 of which are being related to the environment. In this regard, this article aims to extend current literature on this topic by further applying multi-objective optimization methods at the diet level, targeting sustainability. Specifically, this NLP study uses the distance-to-target approach method developed by Abejón et al. [21] and applies it for the first time on diets, in order to simultaneously optimizing three aspects of the sustainability of diets: GHG emissions, nutrition, and economic cost. In order to show the capabilities of the approach, this multi-objective optimization was performed on six different eating patterns within the Spanish context: one based on current food consumption patterns and five alternative diets.

When comparing diets, it is crucial to clearly establish the reference to which they will be compared with. In LCA studies, this reference is provided by the functional unit (FU), which measures the function of the system. While nutrition is the main function of a diet [22], most studies use a mass-based FU (the amount of food consumed by a person). This approach can be misleading since eating less might be more environmentally friendly, while detrimental to health. Some other studies just consider the energy intake to compare diets (isocaloric comparison). However, a healthy diet needs to ensure a minimum intake of macro- and micronutrients, and a maximum intake of certain nutrients, such as saturated fat. In this regard, Batlle-Bayer et al. [23] proposed a method to include this aspect within the FU; and, even, Batlle-Bayer et al. [24] took one step further, by also including food affordability within the FU. They defined the function of a diet as “the intake of the required amounts of energy and nutrients to sustain the body function and daily activity, as well as being affordable”. The current paper uses this approach to be able to compare all diet scenarios.

## 2. Materials and Methods

### 2.1. Pre-Defined Daily Diet Scenarios

This study considered six pre-defined dietary patterns. The first one, the average current consumption (CC) pattern, was defined as the sum of the household food purchases and the food eaten out of home, known as Food Away From Home (FAFH), of an average Spanish citizen in 2018. Data on both types of food consumption were based on the data published by the Spanish Ministry of Agriculture, Fisheries, and Food [25].

Regarding the other five diets, they were designed based on the recommendations given by each of the dietary guidelines:

- The diet based on the National Dietary Guidelines—NDG [26].
- The diet followed the Mediterranean diet pyramid—MED [27].
- The diet followed taking into account ovo-lacto-vegetarian (OLV) recommendations from the Spanish Vegetarian Union
- The diet based on the recommendations for a vegan diet (VEG) provided by the Spanish Vegetarian Union
- The diet followed taking into consideration the Planetary Health (PLH) diet proposed by the EAT-Lancet Commission [1].

To define these alternative diets, the weight serving of the food categories (i.e., red and white meat, processed meat, dairy products, eggs, fruit, vegetables, tubers, legumes, sweets, and beverages) recommended by the guidelines were based on the ones defined by Batlle-Bayer et al. [4]. When no explicit indication of which food products were considered within the recommended food category, the share of these products within the CC pattern was considered. Concerning beverages and sweets, they were just considered when explicitly mentioned in the guidelines. In the case of the VEG diet, the meat substitutes (i.e., tofu) were assumed to be legumes as a protein source. All diets were adjusted to the same amount of energy intake in order to obtain isocaloric daily diets. Based on the energy recommendations of the European Food Safety Authority (EFSA), Spanish population, and the average physical activity level [23]; the weighted average recommended energy intake for an adult Spanish citizen was estimated to be 2228 kcal per day.

### 2.2. Multi-Objective Optimization Programming

For each pre-defined diet, an optimal diet was identified by applying a multi-objective optimization. The most common approach is the traditional ε-constraint approach. This method tackles multi-objective optimization problems by solving the corresponding series of single objective subproblems, where all objectives but one are transformed into constraints. The corresponding solutions, which are not dominated by any other solutions (since an improvement in one objective can only be achieved by accepting a worsening in at least one other objective), are called Pareto optimal points, and the set comprising all these solutions is the Pareto front. Instead of these standard multi-objective optimization methods [21], this study applied a distance-to-target approach, based on Abejón et al. [28], which provides the following advantages: (1) it gives just a single Pareto point instead of a Pareto front of solutions, and (2) it identifies the best way to improve suboptimal solutions by finding minimal projections onto the Pareto front [29]. The Euclidean distance between the individual solutions and the optimization targets can be used as a base [30]. In an n-dimension space (able to represent n optimization objectives), the Euclidean distance (D) is defined by the following equation:

(1) $$D=\overset{n}{\underset{i\;=\;0}{\sum }}{\left|f_{i}\left(x\right)\;-\;g_{i}\right|}^{2}$$

where f_i_(x) is the vector to be optimized and g_i_ the specified target vector. In the current study, a normalized weighted distance D_n_ was employed as the main indicator to identify optimal dietary patterns according to sustainable criteria:

(2) $$D_{n}=\;\frac{\sqrt{\sum_{i\;=\;1}^{n}k_{i}{\left|F_{i}\left(x\right)\;-\;G_{i}\right|}^{2}}}{\sqrt{n}}$$

where F_i_(x) is the normalized vector to be optimized and G_i_ the normalized specified target vector, while k_i_ represents the weighting factor of each objective. When all the objectives are considered equally important, then all the k_i_ factors have a value of 1. Nevertheless, different weighting can be assigned to each objective as a function of the corresponding relative importance, always taking in mind that the sum of all the k_i_ must be equal to the number of objectives (Equation (3)). In order to avoid the lack of consideration of the objectives more distant to target, which could be neglected to minimize D_n_, a limit V_L_ threshold must be defined to maintain all the objectives with a significant contribution (Equation (4)). In this case, the value of V_L_ was fixed at 0.2.

(3) $$\overset{i\;=\;n}{\underset{i\;=\;1}{\sum }}k_{i}=\;n$$

(4) $$k_{i}\;\geq {\;V}_{L}$$

In this way, the definition of Dn implies that the distance values are normalized in the range between 0 (closest to objectives) and 1 (farthest to objectives), so a direct and easily comparable outlook of the results is provided.

Three different sustainable targets were defined. First, the nutritional quality of each diet was evaluated with the Nutrient Rich Diet 9.3 index (NRD9.3) [31]. This index takes into consideration the intake of the 9 encouraging nutrients (protein, fiber, minerals Ca, Fe, Mg and K, and vitamins A, C and E) and the 3 limiting nutrients (saturated fats, added sugar, and Na). The NRD9.3 value can be calculated as the difference between the TNR9 and TNL3 sub-scores (see Equation (5)), where the TNR9 is the sum of percentages of the intake of daily recommended values (RV) of the 9 encouraging nutrients (NRI_i_) and TNL3 is the sum of percentages of the intake of the maximum recommended values (MV) of the 3 limiting nutrients (LNI_i_). As recommended by Drewnowski [32], the intakes were capped to avoid crediting the overconsumption of nutrients to be encouraged. In other words, the intake of a nutrient was set to its RV when the intake of a certain nutrient exceeded it.

(5) $$\mathrm{NRD}9.3\;=\;\mathrm{TNR}9\;-\;\mathrm{TNL}3\;=\;\overset{i\;=\;9}{\underset{i\;=\;1}{\sum }}\frac{{\mathrm{NRIcap}}_{i}}{{\mathrm{RV}}_{i}}\cdot 100\;-\;\overset{i\;=\;3}{\underset{i\;=\;1}{\sum }}\frac{{\mathrm{LNI}}_{i}}{{\mathrm{MV}}_{i}}\cdot 100$$

In addition, a modified NRD9.3_W_ factor was proposed to take into account different relative importance of the different nutrients by assigning different weights (Equation (6)), where kr_i_ and km_i_ represent the weighting factors for encouraging nutrients and limiting nutrients, respectively. Some restrictions were fixed to assure the consideration of all the nutrients (Equations (7)–(10)).

(6) $$\mathrm{NRD}9.3_{W}\;=\;\overset{\;i\;=\;9}{\underset{i\;=\;1}{\sum }}{\mathrm{kr}}_{i}\frac{{\mathrm{NRIcap}}_{i}}{{\mathrm{RV}}_{i}}\cdot 100\;-\;\overset{i\;=\;3}{\underset{i\;=\;1}{\sum }}{\mathrm{km}}_{i}\frac{{\mathrm{LNI}}_{i}}{{\mathrm{MV}}_{i}}\cdot 100$$

(7) $$\overset{i\;=\;9}{\underset{i\;=\;1}{\sum }}{\mathrm{kr}}_{i}\;=\;9$$

(8) $${\mathrm{kr}}_{i}\;\geq \;0.5$$

(9) $$\overset{i\;=\;3}{\underset{i\;=\;1}{\sum }}{\mathrm{km}}_{i}\;=\;3$$

(10) $${\mathrm{km}}_{i}\;\geq \;0.2$$

The environmental target was based on the diet’s GHG emissions. To assess these, the cradle-to-consumption GHG emissions of all the food products within the food basket of a given diet were considered. For each product, a corresponding unitary GHG emissions value (E_i_) (measured as t CO_2_eq./kg of product i, taking into account the model developed by Batlle-Bayer et al. [4]) was provided, and the total emissions of the diet were calculated as the sum of these unitary emissions multiplied by the intake of each product (Q_i_) (measured as kg of product i):

(11) $$\mathrm{GHG}\;=\;\overset{i\;=\;63}{\underset{i\;=\;1}{\sum }}Q_{i}{\;\times \;E}_{i}$$

Likewise, the economic impact of the diets was evaluated, considering the total costs of the food products (TC). For each product, a corresponding unitary cost (C_i_) (measured as €/kg of product i) was assumed and the total costs of the diet were calculated as the sum of these unitary prices multiplied by the intake of each product (Q_i_):

(12) $$\mathrm{TC}\;=\overset{i\;=\;63}{\underset{i\;=\;1}{\sum }}Q_{i}{\;\times \;C}_{i}$$

The three sustainable indicators proposed in this study were normalized in order to obtain values between 0 and 1, which allow the application of a normalized weighted distance approach. On the one hand, the NRD9.3 index was normalized taking into account the maximal theoretical value it can take, which was 900 when all the encouraging nutrients were fully covered, and the limiting nutrients were totally avoided (Equation (13)). On the other hand, the normalization of the environmental and economic indices was based on the comparison with the maximal value resulted from the evaluation of the different diets (Equations (14) and (15)).

(13) $$X_{\mathrm{NUTR}}\;=\;\frac{\mathrm{NRD}9.3}{900}$$

(14) $$X_{\mathrm{ENV}}\;=\;\frac{\mathrm{GHG}}{{\mathrm{GHG}}_{\max}}$$

(15) $$X_{\mathrm{Ec}\;}=\;\frac{\mathrm{TC}}{{\mathrm{TC}}_{\max}}$$

In this case, the multi-objective optimization included the maximization of the nutritional index (X_NUTR target_ = 1) and the simultaneous minimization of the environmental and economic indices (X_ENV target_ = X_EC target_ = 0), which define the target values required to assess the normalized weighted distance D_n_.

It must be remarked that the developed methodology is not absolute and must be adapted to each scenario. The normalization of the economic and environmental indexes, taking into account the product with the highest cost or environmental footprint, is totally senseless since a unique product cannot satisfy the nutritional requirements of a diet.

The optimization process was subject to nutritional constraints. Firstly, the energy content (EN) was limited to be equal to 2228 kcal/day (Equation (16)). Secondly, the intake of at least the daily recommended values of the 9 encouraging nutrients was imposed (Equation (17)). Finally, the maximum recommended values of the 3 limiting nutrients in the diet were defined as limits that must not be exceeded (Equation (18)).

(16) $$\overset{i\;=\;63}{\underset{i\;=\;1}{\sum }}Q_{i}\times EN_{i}=2228$$

(17) $${\mathrm{NRI}}_{i}\;\geq {\;\mathrm{RV}}_{i}$$

(18) $${\mathrm{LNI}}_{i}\;\leq {\;\mathrm{MV}}_{i}$$

Additionally, an acceptability constraint was proposed to limit the deviation from the baseline diet in order to ensure that the food products consumed in the pre-defined diets do not get reduced or increased to extreme levels. It was assumed that the optimized amounts, in grams, must range between threshold values (Tv) around the baseline amount (Q_i base_) as expressed in Equation (19) [20].

(19) $$Q_{i\;\mathrm{base}}\frac{{100\;-\;T}_{V}}{100}\;\leq {\;Q}_{i}\;\leq {\;Q}_{i\;\mathrm{base}}\frac{{100\;+\;T}_{V}}{100}$$

For diets with the baseline nutrient consumption distant to the targets, the definition of the threshold value T_V_ (as proposed in Equation (19)), was not adequate, and in this case, a new range value (N_V_) was applied according to Equation (20).

(20) $$Q_{i\;\mathrm{base}}\frac{10}{100}\;\leq {\;Q}_{i}\;\leq {\;Q}_{i\;\mathrm{base}}{\times \;N}_{V}$$

The definition of minimal threshold values for the weighting of the different objectives is essential to avoid optimizing the problem, just by lack of consideration of the objectives more distant to target. The definition of these minimal threshold values is open to discussion, and the user can define the most convenient value in each case. Moreover, different diets with different nutritional scores can satisfy the nutritional constraints. Therefore, the nutritional score is the most adequate index to take into consideration the real nutritional value of a diet and get it compared with alternatives.

The optimizations were carried out using GAMS software environment to manage the developed non-linear programming (NLP) model using CONOPT3 solver. The General Algebraic Modeling System (GAMS) is a high-level modeling system for mathematical programming and optimization. It consists of a language compiler and a stable of integrated high-performance solvers [33]. Several starting points were tested to demonstrate the robustness of the optimal solution identified. In general, a typical run to solve the mathematic programming problems included more than 1000 single equations and variables, with over 3700 non-zero elements, taking 0.031 central processing unit (CPU) time (s), equivalent to 11 iterations to finish

A Monte Carlo (MC) simulation was performed to analyze the variability of the results given the variation of some of the used coefficients. In order to acquire additional information concerning the influence of the weighting parameters on the nutritional value NRD9.3W of the different diets, a Monte Carlo simulation was carried out to analyze the situation from a stochastic point of view. This simulation was based on the random generation of sets of kri and kmi coefficients (Equation (6)) subject to the corresponding restrictions. These coefficients were applied to the assessment of the NRD9.3W of each diet, leading to a distribution of nutritional values resulted of 1000 different runs. These simulations were useful for visualization of the uncertainty due to the different weighting parameters on the nutritional value and were not used for further statistical analysis [34].

### 2.3. Comparison among Diets

The Batlle-Bayer et al. [24] method was used to compare all pre-defined and optimized diets. This method corrects (indicated with a “c-” in Equation (21)) an environmental impact (i.e., GHG emissions), with three scores:

1. The Residual Income Score (RIS), is based on the concept of the residual income of a diet (RI_diet_): the consumption income (the money left after financial obligations) that remains after diets’ cost. RIS is defined as the ratio between the RI_diet_ (Equation (23)) and the maximal value (RI_max_) (Equation (22)). RI_max_ was set to one, assuming a zero cost, meaning that all the consumption income remains available for other purposes besides food.
2. The energy score (ES), or α (Equations (24) and (25)) account for the energy intake. Since this study evaluates isocaloric daily diets, this is set to 1.
3. The Nutritional Score (NS), is estimated as the ratio between the nutritional quality of a diet (NRD9.3_diet_) and the one of a reference diet (NRD9.3_ref_); in this study, the Planetary Health (PLH) diet.

(21) $${c\text{-}\mathrm{GHG}\;}_{\mathrm{diet}}\;=\;\frac{{\mathrm{GHG}}_{\;\mathrm{diet}}}{\;\mathrm{RIS}*\;\alpha *\;\mathrm{NS}\;}$$

where,

(22) $$\mathrm{RIS}\;=\;\frac{{\mathrm{RI}}_{\mathrm{diet}}}{{\mathrm{RI}}_{\max}}{\;=\;\mathrm{RI}}_{\mathrm{diet}}$$

(23) $${\mathrm{RI}}_{\mathrm{diet}\;}=\;1\;-\;\frac{{\mathrm{Cost}}_{\mathrm{diet}}}{\mathrm{Consumption}\;\mathrm{Income}}$$

(24) $$\alpha \;=\;\mathrm{ES}\;=\;\frac{{\mathrm{DE}}_{\mathrm{diet}}}{{\mathrm{DE}}_{\mathrm{ref}}}{\;\mathrm{if}\;\mathrm{DE}}_{\mathrm{diet}}{\;<\;\mathrm{DE}}_{\mathrm{ref}}$$

(25) $$\alpha \;=\;\frac{1\;}{\mathrm{ES}}{\;\mathrm{if}\;\mathrm{DE}}_{\mathrm{diet}}\;\geq {\;\mathrm{DE}}_{\mathrm{ref}}$$

(26) $$\mathrm{NS}=\;\frac{NRD9.3_{diet}}{NRD9.3_{ref}}$$

### 2.4. Data Collection

Three types of data were collected. First, the nutritional data of all food products were retrieved from the Spanish food composition database [35]. Second, data on annual average prices of all food items, to estimate diet expenditure, were taken from the Spanish Ministry of Agriculture, Fishery, and Food [24]. Third, the GHG emissions related to the production and consumption of all food products were based on Batlle-Bayer et al. [4]. Regarding the consumption income of an average Spanish citizen, it was estimated based on the Spanish Statistical Institute (i.e., INE, using the Spanish acronym).

## 3. Results

### 3.1. Food Intake and Sustainable Factors of the Pre-Defined Diets

**Figure 1:**
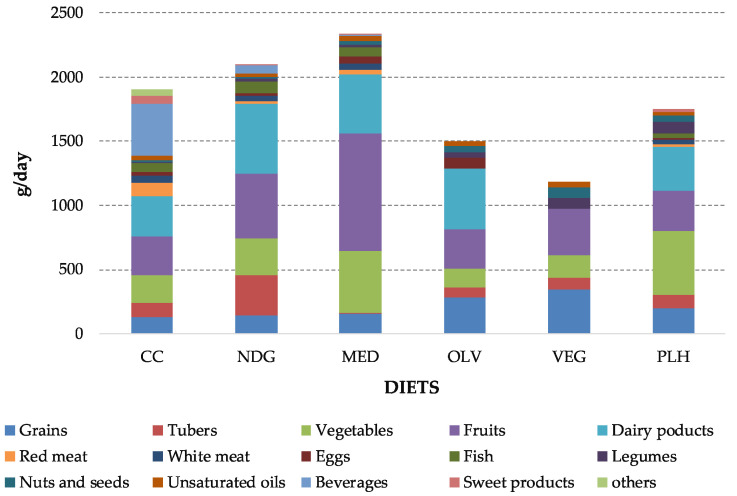
Daily gross food intake (g/day) of different food categories for all pre-defined diets.

The daily gross food intake of the 6 pre-defined diets are shown in Figure 1. The CC diet had the largest intake of red meat (103 g/day) and the lowest consumption of legumes (9 g/day). Its fruit consumption (300 g/day) was similar to the ones of the OLV and PLH diets, and its intake of vegetables (216 g/day) was close to the ones of the OLV and VEG diets. Regarding dairy products, the NDG diet had the largest intake (546 g/day), more than double the amount of CC and PLH diets. The highest intakes of vegetables are found in the MED and PLH diets (485 and 496 g/day), while the largest amount of of grains were consumed in the OLV and VEG diets (285 and 343 g/day, respectively). Concerning nuts, the CC has the lowest value, while the plant-based diets (OLV, VEG, and PLH) had the largest intake. Overall, the MED diet had the largest amount of food intake (2338 g/day), and OLV and VEG had the lowest (1497 and 1183 g/day, respectively).

Table 2 summarizes the three sustainable factors for all pre-defined diets. From the nutritional aspect, the PLH diet presented the best score (680), while the CC diet showed the lowest (498), due to its deficits in fiber and three minerals (i.e., K, Ca, and Mg) and a high intake of saturated fat and salt (Table A1 in Appendix A). In the case of the MED diet, it fulfilled all the relative intakes of encouraging nutrients. In contrast, the NDG diet did not achieve the Mg target (although it was close with 0.975) and vitamin A, and OLV presented deficits for Mg and K. The VEG diet, despite presenting the lowest intake for limiting nutrients (STA, Na, and sugar), had a low intake in terms of protein, K, Vitamin A and, especially, Ca, with a relative intake of 0.357.

**Figure 2:**
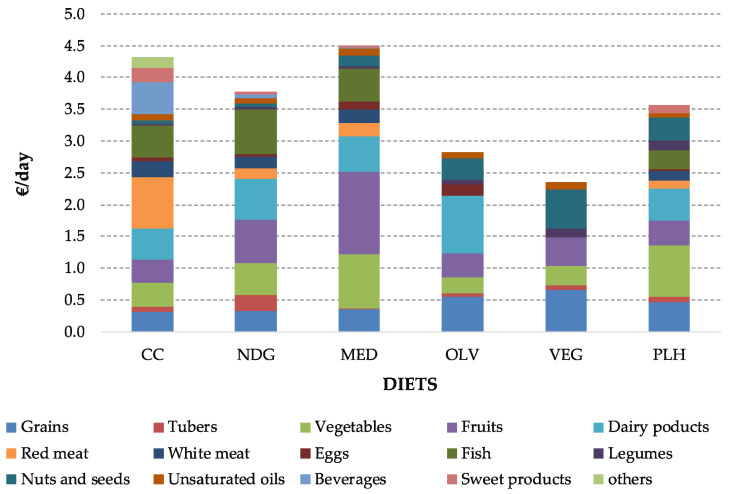
Cost (€/day) of all pre-defined diets.

From an economic perspective, the MED diet was the most expensive one (4.51 €/day), followed by the CC diet. The higher costs of the MED diet can be mainly attributed to vegetables and fruits (Figure 2). Conversely, the VEG diet was the alternative with the lowest costs (2.36 €/day). Finally, in relation to the environmental dimension, the maximal daily GHG emissions corresponded to the CC consumption (4.52 kg CO_2_eq./day), three times higher than the emissions of the VEG diet—the diet with the lowest emissions—due to the great contribution of the food products from animal sources, specifically red meat.

Table 3 shows the distance (D_n_; in Equation (2)) of the six diets, with different normalized weightings (k_i_ for Equation (3)) of the three sustainable aspects. For equally weighting factors (all k_i_ equal to 1), the more plant-based diets were, the lower the distances, and, hence, the more sustainable they were. The shortest distance was computed for the VEG diet, whereas the largest was calculated for the CC: 0.389 vs. 0.826, respectively. When applying unequal normalized weights, the largest distances for all diets (Maximal D_n_ in Table 3) occurred when the least favorable aspect was over-weighted as much as possible, and the other two had the minimal fixed threshold (k_i_ of 0.2). The economic aspect was the least favorable one for the NDG, MED, and VEG diets: while the environmental aspect was the one for the CC and OLV diets. The minimal distances for all diets were obtained when overweighting the most favorable aspect: the nutritional one in all the cases.

### 3.2. Detailed Nutritional Analysis of the Pre-Defined Diets

To further analyze the nutritional status of the pre-defined diets, a modified NRD9.3_W_ factor was proposed (Equation (6)). Instead of considering that all nutrients are equally relevant (as done in NRD9.3), the NRD9.3_W_ takes into account the different relative importance of the different nutrients by assigning different weights. This new nutritional factor was subject to a scenario analysis in order to see the influence of the different weights of each nutrient.

**Figure 3:**
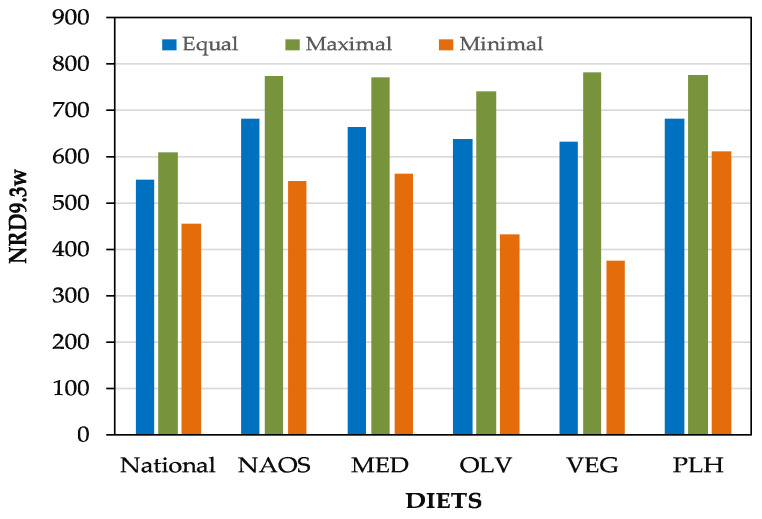
Nutritional index (NRD9.3_w_) of the different pre-defined diets subject to variable weighting factors of the nutrients.

Figure 3 shows the values of NRD9.3_w_ under different scenarios: (1) equal relevance of the 12 nutrients, (2) maximization of the relevance of the nutrients with best nutritional performance, and (3) minimization of the relevance of the nutrients with worst nutritional performance. As previously identified, the CC diet is the poorest nutritional option for both equal and maximal cases. However, when the minimal values were compared, the VEG diet resulted in the worst option (374 vs. 448 for CC). This was due to the low relative intake of certain encouraging nutrients, which implied a great handicap when their weights were over-represented. In contrast, since the VEG diets showed a very low intake of limiting nutrients, this option took advantage of the high relative importance of these factors and appeared as the highest maximal value (780), slightly higher than the PLH (776).

**Figure 4:**
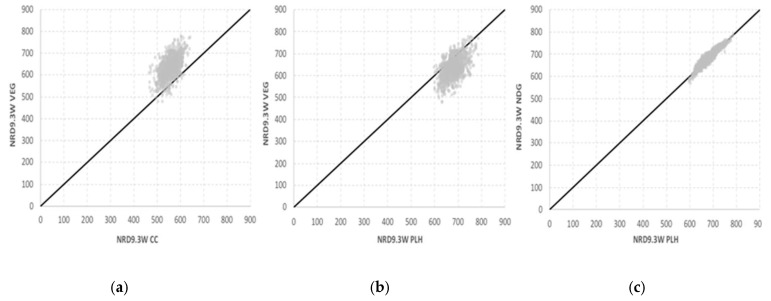
Direct comparison of the nutritional indices obtained from the Monte Carlo analysis: (**a**) VEG diet vs. CC diet; (**b**) VEG diet vs. PLH diet; and (**c**) NDG diet vs. PLH diet.

**Figure A1:**
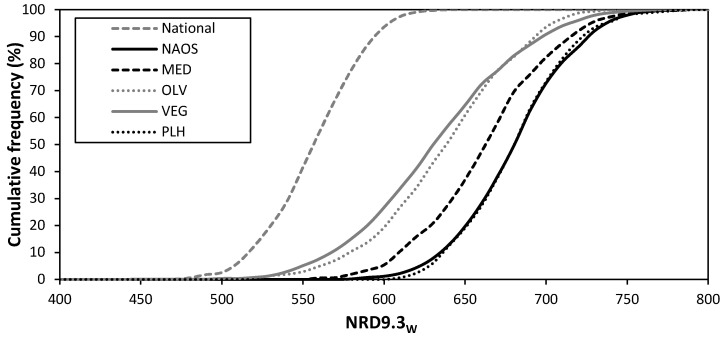
Cumulative frequency of the NRD9.3w values of the different pre-defined diets in the Montecarlo analysis.

The influence of the weighting parameters on the nutritional value NRD9.3_W_ of the different diets was investigated in a Monte Carlo simulation. Aleatory values for kr_i_ and km_i_ coefficients were applied to the assessment of the NRD9.3_W_ of each diet, leading to a distribution of nutritional values (Figure A1 in Appendix A). The CC diet consistently fell out below the nutritional levels achieved by the alternative diets. As an illustrative example, the direct comparison of the CC versus the VEG diet (which had been identified as the one that could be represented by the lowest nutritional values under unfavorable weighting conditions) is shown in Figure 4a. The most frequent NRD9.3_W_ values for CC ranged between 450 and 650, but those for the VEG diet varied from 500 to 750. Even when the highest values of the CC diet were attained, the corresponding weighting coefficients resulted in higher nutritional levels for the VEG diet, with very few points below the diagonal line in the graph. Among the alternative diets, the PLH diet was the optimal according to the nutritional index defined, since only the VEG (Figure 4b) and the NDG (Figure 4c) diets were able to attain higher NRD9.3_W_ values in a little number of scenarios.

### 3.3. The Optimal Diet without Acceptability Restrictions

The initial optimization approach was to identify the optimal diet without applying any acceptability constraint. Under these circumstances, the intake of food products was totally allowed (not subject to restrictions) to choose as much as necessary of a given food product or to eliminate a product from the market basket completely.

By minimizing the distance under equal weighting of the three sustainable dimensions, the resulting diet accounted for few food products (seven in total; Table A2), it had a NRD9.3 of 820, it cost 1.54 €, it emitted about 1.30 kg CO_2_eq. (Table A2), and its distance (D_N_) was 0.27. This D_N_ was decreased to 0.13 when the nutritional aspect was over weighted. The resulting diet was comprised of only four food products: milk, legumes, sunflower oil, and other vegetables (Table A2).

In summary, optimizing without acceptability constraints resulted in low diverse diets with few food products and food categories. This means that these conditions should not be considered as representative of a real, healthy diet. However, they may be used as a benchmark to identify low-budget diets when substantial economic constraints affect vulnerable population groups [36], in which citizens rely on a minimal amount of food products to subsist.

**Figure A2:**
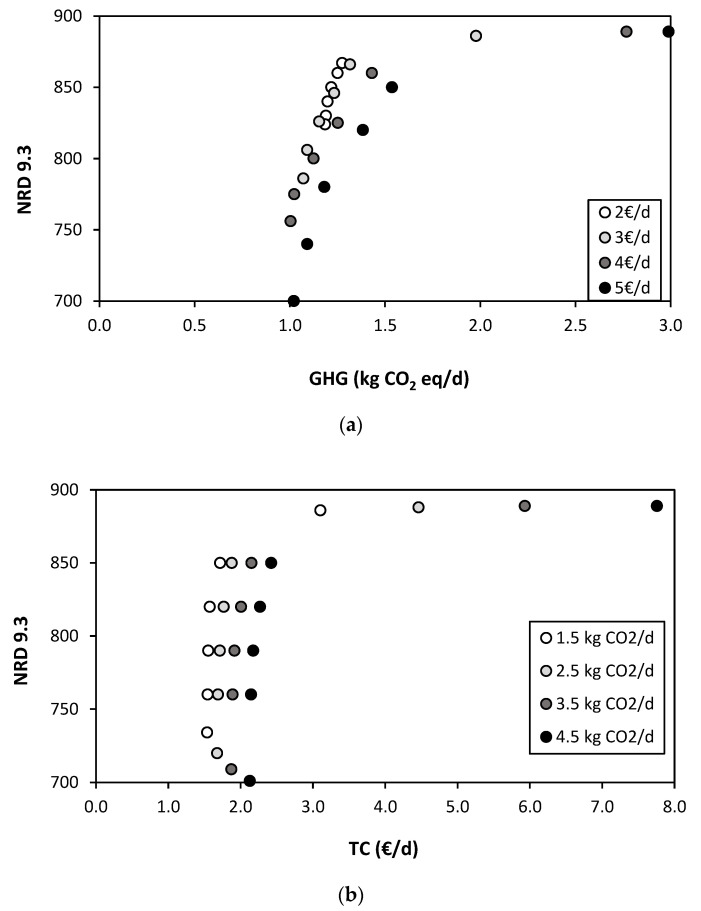

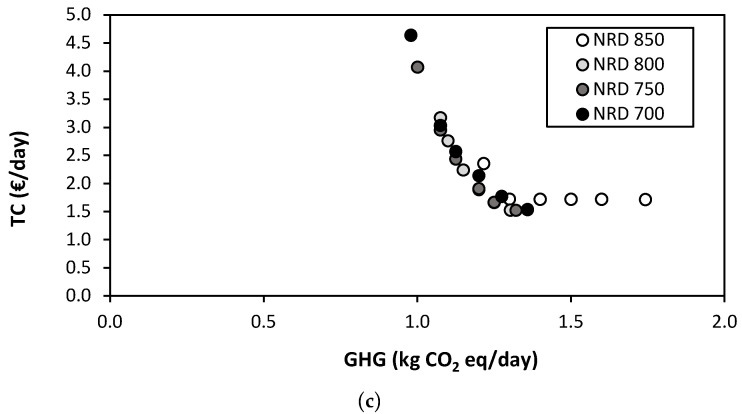
Pareto fronts of the multi-objective optimization of diets without constraints: (**a**) Constant economic index, (**b**) Constant environmental index, (**c**) Constant nutritional index.

Despite the use of a distance-based approach to investigate the multi-objective optimization of diets, a traditional ε-constraint approach was also implemented. This way, the Pareto fronts for the three objectives without restrictions on food intake were obtained (Figure A2 in Appendix A). The analysis of the Pareto fronts revealed that the relaxation in one objective allowed the achievement of more extreme values in the other objectives, but the distance to the targets was not significantly reduced and, in some cases, it increased. Focusing on the Pareto fronts obtained for defined economic costs represented in Figure A2 in Appendix A, the comparison of the result calculated when the cost was limited to 2 and 5 €/day confirmed the explained trend. While the limit of 5 €/day allowed more extreme values, in the other objectives when compared to 2 €/day (the NRD9.3 value increased from 867 to 889 and the GHG emissions were reduced from 1.185 to1.021), the distance of the front to the target (NRD9.3 = 900 and GHG = 0) clearly increased. In addition, the total distance must consider the clear disadvantage in the economic dimension that a 5 €/day assumption implied when compared to only 2 €/day.

### 3.4. Optimal Diets with Acceptability Constraints

#### 3.4.1. Variability Margins (T_v_)

To optimize all pre-defined diet scenarios with acceptability restrictions, the first step was to determine the minimal variability margins (Equation (19)), to ensure that all optimized diets fulfilled the nutritional requirements and represented their baseline dietary patterns. Table 4 summarizes all the variation thresholds (T_v_) and the nutrients that restricted the diets. In the case of the VEG diet, the threshold value N_V_ was used, instead of T_V_, since it was the diet with the most distant nutrient index to its target (relative intake of Ca was only 0.364).

The value of N_V_ for the VEG diet was 7, in order to fulfill the Ca requirement without forcing the Na limit. This balance between Ca and Na was also the limiting restriction for the CC diet, which needed a 54% margin to satisfy the requirements, a percentage similar to that of the OLV diet (49%). However, for the latter, the restriction was the balance between K and saturated fat. Although the MED diet only needed to reduce the saturated fat intake to fulfill the requirements, a 23% margin was necessary. Finally, only a 3% margin was necessary to reduce the saturated fat intake in the PLH diet.

**Figure 5:**
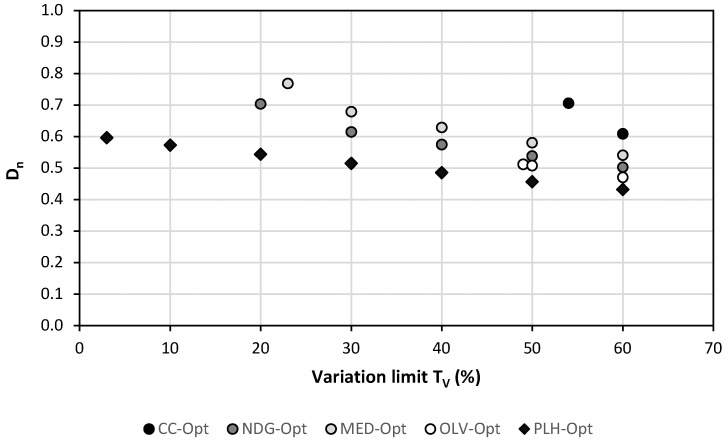
Distance (Dn) for optimal diets with different variation limits.

**Figure A3:**
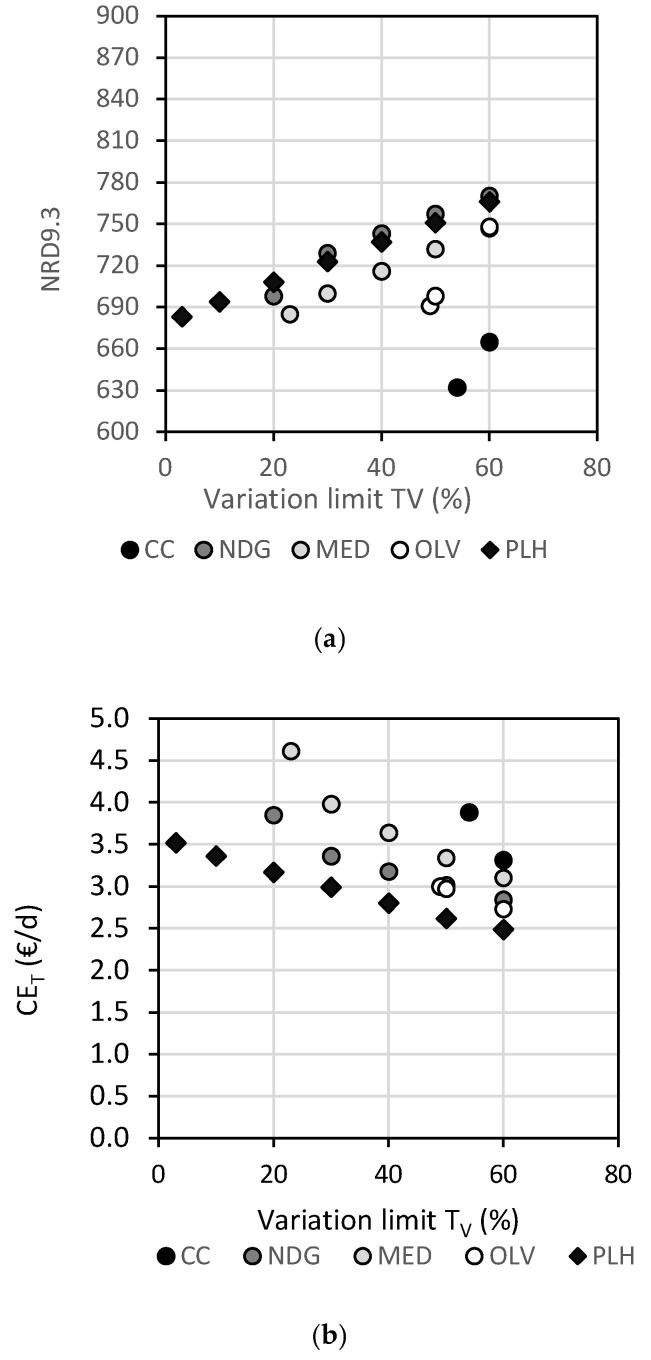

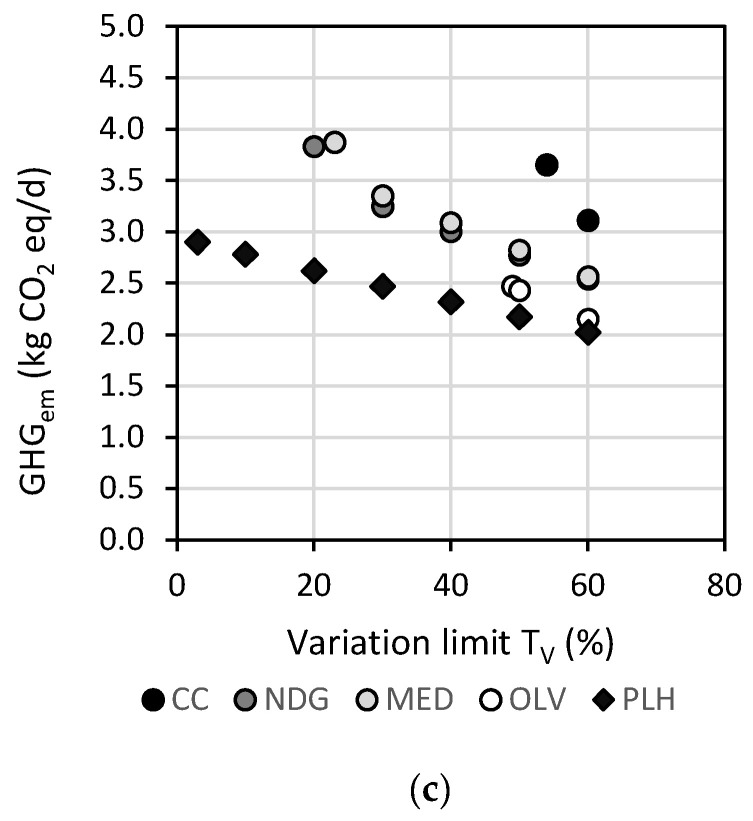
Optimization of the diets according to nutritional (**a**), economic (**b**), and environmental (**c**), targets as functions of the acceptability variation limits.

Figure 5 depicts the distance (D_n_) of the optimized diets with different maximal margins of variations (T_v_) (see Figure A3 in Appendix A for the three sustainable factors). The VEG diet was not included since it required the definition of N_V_ values, and the corresponding results cannot be easily compared. In fact, due to the minimal T_V_ values required to fulfill the nutritional restrictions imposed, the comparison of all the diets was only possible for values above 54%. The definition of higher margins of variation allowed the diets to improve the optimal values in all the objectives. With a T_V_ value of 60%, the distances decreased for all diets between 13 and 32%. PLH appeared to be the option with the lowest distance (0.432).

#### 3.4.2. Food Intake of the Optimized Diets and Their Corrected GHG Emissions

**Figure 6:**
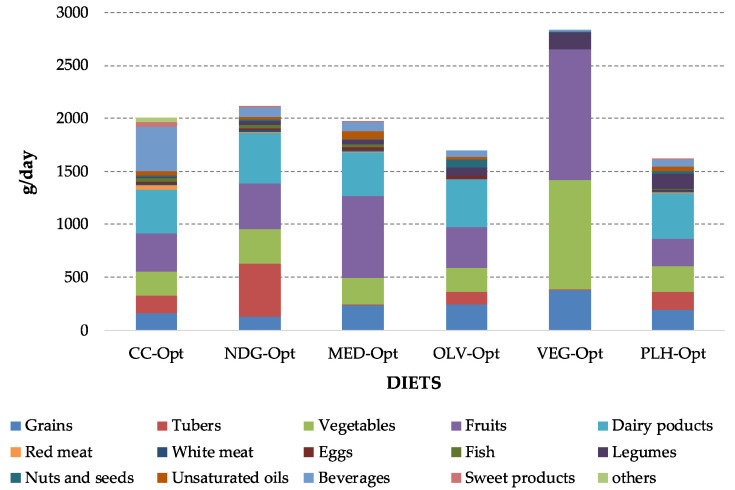
Daily gross food intake (g/day) of different food categories for all optimized diets.

Figure 6 shows the gross food intake (g/day) for all the optimized diets (for more detailed values see Table A4 in Appendix A). Although the optimal intake of the different foods depended on the optimization target, the consumption of red and white meat as well as fish and eggs were reduced in all cases where consumed. Instead, other products were promoted to be taken in higher quantities for all the objectives, such as bread, legumes, vegetables, and tubers.

The sustainable factors (price, NRD9.3, and GHG emissions) of all diets are summarized in Table 5. The ones of the CC-Opt, MED-Opt, NDG-Opt, and PLH-Opt diets were all improved compared with the ones of their corresponding pre-defined scenarios. However, different outcomes were found for the optimized vegan diet (VEG-Opt). While its nutritional value increased (becoming the most nutritional diet among all the dietary scenarios), the GHG emissions (1.81 kg CO_2_eq./day) and its cost (4.28 €/day) were higher than the ones of the baseline VEG diet (1.41 kg CO_2_eq./day and 2.36 €/day). In the case of the OLV-Opt diet, its environmental and nutritional factors improved, but its cost is slightly higher than the one of the OLV diet.

**Figure 7:**
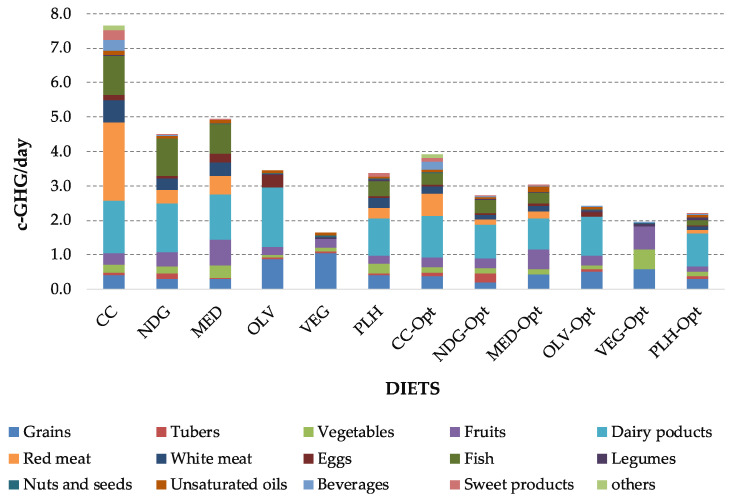
Corrected GHG emissions of all diet scenarios.

To compare sustainability among diets is challenging due to its multidimensionality. In this regard, this study applied the methodology of Batlle-Bayer et al. [24], which uses two scores (i.e., RIS and NS) to correct GHG emissions, in order to consider the nutritional and economic aspects when comparing the environmental performance of diets. Once GHG emissions are corrected, the VEG-opt has the best performance (1.92 c-kg CO_2_eq./day^−1^) among the optimized diets. Nevertheless, the VEG diet has still the lowest corrected emissions (1.65 c-kg CO_2_eq./day^−1^), due to the lower GHG emissions and the larger affordability. However, the insufficient intake of protein, Ca, and vitamin A should be kept in mind (Table A1 in Appendix A). Regarding the remaining diets, PLH-Opt and OLV-Opt also show relatively low corrected emissions, whereas the optimized CC-Opt diet had the highest value (3.90 c-kg CO_2_/day), being the dairy products and red meat the largest contributors (Figure 7).

## 4. Discussion and Conclusions

The identification of optimal diets, according to sustainable patterns, is a complex task. The definition provided by FAO already offers a glimpse of this complexity, since a sustainable diet must be “protective and respectful of biodiversity and ecosystems, culturally acceptable, accessible, economically fair and affordable; nutritionally adequate, safe and healthy; while optimizing natural and human resources” [37]. Although increased attention has been paid to sustainable diets, it is still unclear how the different components of sustainable diets must be defined, measured, and prioritized to achieve optimal results [38]. Hence, the search for optimal sustainable diets implies simultaneous optimization of their environmental, economic, health (nutrition), and other socio-cultural dimensions [39], and partial optimization should be avoided [40,41]. The intrinsic relations among these different aspects may result in opposing objectives and, therefore, only trade-offs can give the best solutions.

The methodology proposed in this work aggregates the nutritional, economic, and environmental aspects of a diet in a unique indicator based on the distances to the objectives. This methodology is universal, but not absolute, and can be adapted to different scenarios. In this regard, this article applies the distance-to-target approach for multi-objective non-linear optimization of 6 dietary scenarios. While this approach is new at the diet level, it has been used in other sectors. For instance, Limleamthong and Guillén-Gosálbez [29] applied this approach to optimize the British electricity mix, according to economic and environmental criteria. As well, Asante et al. [42] employed a multi-objective linear optimization based on distances to obtain a ranking of the barriers to implement renewable energies in developing countries. In the case of Oliveira et al. [43] and d’Inverno and de Witte [44], both developed composite indicators based on distances, and Wheeler et al. [45] proposed a post-optimal analysis of the obtained Pareto points based in distances in order to select the solution that best fulfills the decision-makers’ preferences. Within the food sector, Abejón et al. [28] used a distance-to-target approach based on the definition of a normalized weighted distance to provide practical and effective optimization guidelines by measuring the magnitude towards the sustainable quantitative targets in the minimization of economic and nutritional costs associated to food loss and waste. This method has been adapted here to diets, and three objectives were considered simultaneously: the minimization of both the GHG emissions and the cost of the diet, and the maximization of the diet’s nutritional value.

For all the optimized diets, the intake of animal protein, namely red meat, significantly decreased. This finding is coherent with previous studies, in which French [46] and Dutch [47] diets were optimized. Regarding diet affordability, all the optimized diets were more affordable than the current equivalents, except for the vegan (VEG) diet, which did not show any significant savings. In the case of the optimized planetary health (PLH) diet, it was the cheapest among the optimized diets, and its value is close to the cost of the global median PLH diet reported by Hirvonen et al. [48] at 2.84 US $/day. Nevertheless, our study has not considered plant-based processed foods, such as tofu or seitan, which may increase the cost, and environmental emissions, of plant-based diets. Hence, further exhaustive investigation that considers more diverse food products and their prices are required. Besides, the consideration of micronutrients that have not been taken into account in this work, such as starch, vitamin B12 of natural sugars could result in different optimized diets. For instance, the consumption of potatoes (rich in starch) or fruits (rich in natural sugars) might be reduced as a consequence of a more precise scenario regarding micronutrients. Finally, concerning GHG emissions (both the GHG and the c-GHG), the optimized diets show lower values than the respective pre-defined ones except for the VEG, which increases slightly in both indicators. Thus, reductions in c-GHGs are in the range of 46% for CC and 30% for OLV. Despite the slight increase, the VEG and VEG-opt present the best values for both GHG and c-GHG (1.41 kg CO_2_eq./day and 1.65 c-kg CO_2_eq./day, and 1.65 kg CO_2_eq./day and 1.92 c-kg CO_2_eq./day, respectively) comparing to the worst performer that is CC.

The optimization of this study used a 54% variation range. This value for the departure from the actual diet is larger compared to some studies (i.e., 20% for [22]), similar to others, such as Song et al. [49] or Broekema et al. [47], with variation ranges between 33 and 150%, and smaller than the global median departure value of 177% reported by Chaudhary and Krishna [20]. Nevertheless, the key message of these departure values is the fact that current food habits are far from sustainable.

The analysis of this study should be enlarged by adding other key elements when assessing environmental impacts. First, it must be noted that food losses and waste are important drivers of the food-related environmental impacts. In fact, certain studies have alerted that the shift to healthier diets tends to generate higher amounts of food losses and waste [50,51]. Second, other resources-based metrics, such as land use or water footprint, both critical in the agricultural and livestock sector, would allow identifying the trade-offs between environmental impacts, as well as determining the feasibility of the resulting changes in the dietary patterns based on the resources needed. In other words, large-scale diet changes that imply an additional amount of land for cultivation may be constrained by a lack of additional available farmable land [52]. Third, the use of pesticides should also be considered in further analysis. If more plant-based products are recommended, the possible increase of their production may enhance pesticide application, with its potentially detrimental impact to biodiversity.

The optimization studies, as performed here, are crucial to design sustainable diets and highlight the importance of guidelines to write more concisely food recommendations. Unclear advice may lead to undesired results. Moreover, further investigation on understanding the drivers behind consumers’ food habits and how they can accept/adopt changes in their daily eating patterns is essential. Lehikoinen and Salonen [53] found that the combination of hedonistic factors and the environmental benefits were crucial aspects to ensure that Finnish consumers would shift to plant-based diets. In this regard, research on how Spanish consumers perceive sustainable diet transitions and their drivers for change is needed. This will allow to better estimate the potential environmental, nutritional, and economic benefits of diet shifting.

## Figures and Tables

**Table 1:** Definition of objective functions of different types of optimization methods used to design sustainable diets. e, environmental impact; x, amount (mass) of a food product; n, total amount of food products; opt, optimal diet; base, baseline diet; c, cost; i: food item; j, environmental impact.

Optimization Method	Objective Function	Reference
Linear programming (LP)	$\mathrm{Min}\;f=\overset{n}{\underset{i=1}{\sum }}e_{i}x_{i}{=e}_{1}x_{1}{+\ldots +e}_{n}x_{n}$	[13]
Multi-objective target programming (MTP)	$\mathrm{Min}\;f=\frac{1}{2}\overset{m}{\underset{j=1}{\sum }}\frac{(\;\sum_{i=1}^{n}e_{\mathrm{ji}}x_{\mathrm{opt},i}{)\;-\;e}_{\mathrm{base},j\;}}{e_{\mathrm{base},j}}\;+\frac{1}{2}\;\frac{(\;\sum_{i=1}^{n}c_{i}x_{\mathrm{opt},i}{)\;-\;c}_{\mathrm{base}\;}}{c_{\mathrm{base}}}$	[17]
Non-linear programming (NLP)	$\mathrm{Min}\;f=\overset{n}{\underset{i=1}{\sum }}{\left(\;\frac{x_{\mathrm{opt},i}\;{-\;x}_{\mathrm{base},i}}{x_{\mathrm{base},i}}\right)}^{2}$	[20]

**Table 2:** Nutritional, economic, and environmental indexes of the pre-defined diets.

	CC	NDG	MED	OLV	VEG	PLH
**NRD9.3** (−)	498	679	662	637	632	680
**TC** (€/day)	4.32	3.77	4.51	2.83	2.36	3.56
**GHG** (kg CO_2_eq./day)	4.52	3.93	4.07	2.91	1.41	2.95
**X_NUTR_**	0.611	0.754	0.736	0.708	0.702	0.756
**X_ENV_**	1.000	0.869	0.903	0.644	0.312	0.653
**X_EC_**	0.947	0.827	1.000	0.621	0.518	0.781

**Table 3:** Distances (Dn) of the pre-defined diets (including maximal and minimal values).

Weighting	Distance (D_n_)					
	CC	NDG	MED	OLV	VEG	PLH
Equally weighted	0.826	0.707	0.793	0.543	0.389	0.604
Maximal D_n_	0.968	0.840	0.962	0.625	0.495	0.749
(overweighted aspect)	ENV	ENV	EC	ENV	EC	EC
Minimal D_n_	0.508	0.385	0.426	0.357	0.318	0.348
(overweighted aspect)	NUTR	NUTR	NUTR	NUTR	NUTR	NUTR

**Table 4:** Minimal acceptability variation limits required for each diet to fulfill the nutritional restrictions.

Diet	Limiting Nutrient	Required T_V_ or N_V_
CC-opt	Ca/Na	T_V_ = 54%
NDG-opt	Vit A/STA	T_V_ = 20%
MED-opt	STA	T_V_ = 23%
OLV-opt	K/STA	T_V_ = 49%
VEG-opt	Ca/Na	N_V_ = 7
PLH-opt	STA	T_V_ = 3%

**Table 5:** Sustainable factors and scores to estimate the corrected GHG emissions (c-GHG).

Diet Scenarios		Sustainable Factors			Residual Income Score (RIS)	Nutritional Score (NS)	c-GHG
		Price (€/day)	Nutrition (NRD9.3)	GHG (kg CO_2_eq./day)			
**Optimized**	VEG-Opt	4.28	748	1.81	0.85	1.10	1.92
	PLH-Opt	2.57	717	2.12	0.91	1.06	2.21
	OLV-Opt	2.88	690	2.18	0.90	1.01	2.38
	NDG-Opt	2.92	728	2.61	0.90	1.07	2.71
	MED-Opt	3.17	684	2.71	0.89	1.01	3.02
	CC-Opt	3.33	620	3.15	0.89	0.91	3.90
**Pre-defined**	VEG	2.36	632	1.41	0.92	0.93	1.65
	PLH	3.56	680	2.95	0.88	1.00	3.36
	OLV	2.83	637	2.91	0.90	0.94	3.44
	NDG	3.77	679	3.93	0.87	1.00	4.51
	MED	4.51	663	4.07	0.85	0.97	4.94
	CC	4.32	498	4.52	0.85	0.73	7.28

## Appendix A

**Table A1:** TNR9, TNÑ3, and NRD9.3 of the pre-defined diets.

	Nutrient	CC	NDG	MED	OLV	VEG	PLH
**TNR9**	Proteins	100.0	100.0	100.0	100.0	82.3	100.0
	Fiber	93.5	100.0	100.0	100.0	100.0	100.0
	K	94.4	100.0	100.0	78.2	72.3	100.0
	Ca	91.0	100.0	100.0	100.0	35.7	100.0
	Fe	100.0	100.0	100.0	100.0	100.0	100.0
	Mg	97.2	97.5	100.0	95.0	100.0	100.0
	Vitamin A	100.0	89.1	100.0	100.0	64.5	100.0
	Vitamin C	100.0	100.0	100.0	100.0	100.0	100.0
	Vitamin E	100.0	100.0	100.0	100.0	100.0	100.0
**TNL3**	Saturated Fat (SAT)	135.8	107.0	120.8	128.9	58.4	102.5
	Na	152.9	67.3	83.3	65.6	56.6	84.5
	Added sugar	89.2	32.8	33.9	41.7	9.9	33.4
**NRD9.3**		**498.1**	**679.4**	**662.0**	**637.0**	**631.8**	**679.6**

**Table A2:** Food intake (g/day) of four optimized diets without restrictions.

Product Intakes G_i_ (g/day)	Minimal D_n_ (Equally Weighted)	Minimal D_n_ (Inequally Weighted)
Canned anchovies		
Milk	509.8	475.8
Rice		
Legumes	415.0	547.2
Sunflower oil	1.5	4.3
Margarine	54.2	
Potatoes	39.0	
Other vegetables	11.5	278.6
Citrus fruits	238.3	
Peaches		
Olives		
Salt		

**Table A3:** Factors for all optimized diet scenarios without restrictions.

	Minimal D_n_ (Equally Weighted)	Minimal D_n_ (InequallyWeighted)
**Sustainability factors**		
NRD9.3 (−)	820	854
GHG (kg CO_2_eq./day)	1.297	1.234
TC (€/d)	1.54	1.76
**Normalized factors**		
X_NUTR_	0.911	0.949
X_ENV_	0.288	0.274
X_EC_	0.349	0.399
D_n_	**0.266**	**0.133**

**Table A4:** Food intake (g/day) of all pre-defined and optimized diets.

Food Products Intake (g/day)	Pre-Defined Diets						Optimized Diets (Minimal D_N_)					
	CC	NDG	MED	OLV	VEG	PLH	CC-Opt	NDG-Opt	MED-Opt	OLV-Opt	VEG-Opt	PLH-Opt
Eggs	25.0	15.8	58.6	82.5	0.0	12.7	10.0	17.1	23.4	33.0	0.0	5.1
Beef	17.1	5.2	6.7	0.0	0.0	4.7	6.8	2.1	2.7	0.0	0.0	1.9
Chicken	55.2	41.0	45.9	0.0	0.0	33.3	22.1	16.4	18.4	0.0	0.0	13.3
Rabbit	3.9	2.9	3.2	0.0	0.0	2.3	1.6	1.2	1.3	0.0	0.0	0.9
Lamb	5.0	1.5	2.0	0.0	0.0	1.4	2.0	0.6	0.8	0.0	0.0	0.6
Pork	31.9	11.0	14.1	0.0	0.0	4.7	12.8	4.4	5.6	0.0	0.0	1.9
Processed meat	48.7	5.0	6.4	0.0	0.0	5.2	19.5	2.0	2.6	0.0	0.0	2.1
Hake	22.3	30.6	22.5	0.0	0.0	12.9	8.9	12.2	9.0	0.0	0.0	5.2
Pilchard	4.7	6.4	4.7	0.0	0.0	2.7	7.5	2.6	1.9	0.0	0.0	1.1
Tuna	1.8	2.4	1.8	0.0	0.0	1.0	0.7	1.0	0.7	0.0	0.0	0.4
Atlantic mackerel	1.2	1.6	1.2	0.0	0.0	0.7	0.5	0.6	0.5	0.0	0.0	0.3
Salmon	4.0	5.5	4.1	0.0	0.0	2.3	1.6	2.2	1.6	0.0	0.0	0.9
Hake (frozen)	8.3	11.5	8.4	0.0	0.0	4.8	3.3	4.6	3.4	0.0	0.0	1.9
Mussels	5.2	7.2	5.3	0.0	0.0	3.0	2.1	2.9	2.1	0.0	0.0	1.2
Squid and octopus	5.0	6.9	5.1	0.0	0.0	2.9	2.0	2.8	2.0	0.0	0.0	1.2
Prawns and shrimps	1.6	2.2	1.6	0.0	0.0	0.9	0.6	0.9	0.6	0.0	0.0	0.4
Squid and octopus (frozen)	2.6	2.8	2.1	0.0	0.0	1.2	1.0	1.1	0.8	0.0	0.0	0.5
Prawns and shrimps (frozen)	6.1	9.1	6.7	0.0	0.0	3.8	2.4	3.6	2.7	0.0	0.0	1.5
Tuna	5.6	7.7	5.6	0.0	0.0	3.2	2.2	3.1	2.2	0.0	0.0	1.3
Mussels	0.7	0.9	0.7	0.0	0.0	0.4	0.3	0.4	0.3	0.0	0.0	0.2
Anchovies	0.9	1.2	0.9	0.0	0.0	0.5	0.4	0.5	0.4	0.0	0.0	0.2
Milk	214.1	422.3	362.0	216.8	0.0	247.2	342.6	424.7	366.5	346.9	0.0	394.2
Shakes	10.6	0.0	0.0	0.0	0.0	0.0	17.0	0.0	0.0	0.0	0.0	0.0
Ice cream	9.6	0.0	0.0	0.0	0.0	0.0	3.8	0.0	0.0	0.0	0.0	0.0
Yoghurt	60.5	109.0	89.1	225.8	0.0	69.8	24.2	43.6	35.6	90.3	0.0	27.9
Butter	0.9	0.0	0.0	0.0	0.0	0.0	0.4	0.0	0.0	0.0	0.0	0.0
Fresh cheese	14.5	10.9	9.0	22.7	0.0	16.8	15.7	4.4	3.6	9.1	0.0	6.7
Semi-hard cheese	7.0	3.3	4.3	11.0	0.0	8.1	2.8	1.3	1.7	4.4	0.0	3.2
Hard cheese	1.6	0.7	1.0	2.4	0.0	1.8	2.6	0.3	0.4	1.0	0.0	0.7
Bread	103.3	111.3	133.6	109.7	132.2	162.5	114.0	113.5	213.8	175.5	358.7	177.6
Rice	11.4	16.1	11.2	87.8	105.7	18.0	18.2	6.4	17.9	35.1	10.6	7.2
Pasta	12.0	15.5	10.4	87.8	105.7	18.9	19.2	6.2	4.2	35.1	10.6	7.6
Biscuits	35.6	5.0	6.4	0.0	0.0	18.5	14.2	2.0	2.6	0.0	0.0	7.4
Cereals	4.9	0.7	0.9	0.0	0.0	2.5	7.8	0.3	0.4	0.0	0.0	1.0
Tablet	4.4	0.6	0.8	0.0	0.0	2.3	1.8	0.2	0.3	0.0	0.0	0.9
Snack choco	1.7	0.2	0.3	0.0	0.0	0.9	0.7	0.1	0.1	0.0	0.0	0.4
Cacao powder	4.6	0.6	0.8	0.0	0.0	2.4	7.4	1.0	1.3	0.0	0.0	1.0
Sugar	11.9	0.0	0.0	0.0	0.0	0.0	19.0	0.0	0.0	0.0	0.0	0.0
Legumes	9.3	20.8	21.4	45.2	81.6	85.9	14.9	33.3	34.2	72.3	155.4	137.4
Olive oil	25.2	22.5	28.9	31.0	37.3	17.4	21.0	9.0	46.2	27.1	3.7	27.8
Sunflower oil	12.1	10.8	13.9	0.0	0.0	8.4	19.4	17.3	22.2	0.0	0.0	13.4
Margarine	2.1	0.0	0.0	0.0	0.0	0.0	0.8	0.0	0.0	0.0	0.0	0.0
Potatoes	107.4	316.8	7.4	75.2	90.6	104.8	171.8	506.9	11.8	120.3	9.1	167.7
Tomatoe	41.4	153.8	264.4	30.9	37.2	105.1	16.6	246.1	162.0	49.4	30.2	48.2
Lettuce	12.5	103.1	177.2	13.3	16.0	31.7	5.0	41.2	70.9	5.3	110.7	14.6
Others	123.5	25.0	43.1	105.4	127.0	359.0	188.3	40.0	17.7	167.4	889.0	180.8
Citric	135.7	149.7	264.0	136.5	164.4	142.0	217.1	239.5	422.4	218.4	1150.8	190.6
Bananas	55.1	164.5	290.2	55.4	66.7	57.6	88.2	109.9	202.9	88.6	6.7	23.0
Apples	77.9	66.8	117.8	78.4	94.4	81.5	31.2	26.7	47.1	31.4	9.4	32.6
Peach	24.3	94.5	166.7	24.4	29.4	25.4	9.7	37.8	66.7	39.0	2.9	10.2
Olives	7.5	27.5	74.3	7.5	9.1	7.8	12.0	11.0	29.7	12.0	63.7	3.1
Nuts	8.5	8.5	22.9	47.2	85.3	52.4	13.6	13.6	15.3	75.5	8.5	28.4
Tomato products	38.8	0.0	0.0	0.0	0.0	0.0	15.5	0.0	0.0	0.0	0.0	0.0
Gazpacho	13.8	0.0	0.0	0.0	0.0	0.0	5.5	0.0	0.0	0.0	0.0	0.0
Fabada	24.5	0.0	0.0	0.0	0.0	0.0	28.6	0.0	0.0	0.0	0.0	0.0
Ketchup	8.0	0.0	0.0	0.0	0.0	0.0	3.2	0.0	0.0	0.0	0.0	0.0
Coffee	6.0	0.0	0.0	0.0	0.0	0.0	2.4	0.0	0.0	0.0	0.0	0.0
Wine	32.5	5.0	1.3	0.0	0.0	0.0	13.0	2.0	0.5	0.0	0.0	0.0
Beer	53.9	9.6	7.1	0.0	0.0	0.0	86.2	3.8	2.8	0.0	0.0	0.0
Water	29.6	9.4	0.0	0.0	0.0	0.0	62.0	62.0	62.0	62.0	0.0	0.0
Juice	155.0	0.0	0.0	0.0	0.0	0.0	47.4	3.8	0.0	0.0	15.5	62.0
Soft drinks	130.3	38.2	0.0	0.0	0.0	0.0	208.5	15.3	24.5	0.0	0.0	0.0
Salt	3.4	0.0	0.0	0.0	0.0	0.0	1.4	0.0	0.0	0.0	0.0	0.0
**TOTAL**	**1904.0**	**2101.0**	**2337.8**	**1496.6**	**1182.7**	**1753.3**	**2003.0**	**2105.3**	**1970.4**	**1699.3**	**2835.6**	**1617.4**
